# Estrogen, progesterone, and human epidermal growth factor receptor 2 discordance between primary and metastatic breast cancer

**DOI:** 10.1007/s10549-020-05746-8

**Published:** 2020-07-01

**Authors:** Vincent Walter, Chiara Fischer, Thomas M. Deutsch, Catherine Ersing, Juliane Nees, Florian Schütz, Carlo Fremd, Eva-Maria Grischke, Peter Sinn, Sara Y. Brucker, Andreas Schneeweiss, Andreas D. Hartkopf, Markus Wallwiener

**Affiliations:** 1 Department of Obstetrics and Gynecology, University Hospital Tuebingen, Calwerstraße 7, 72076 Tuebingen, Germany; 2 Department of Obstetrics and Gynecology, University Hospital Heidelberg, Im Neuenheimer Feld 440, 69120 Heidelberg, Germany; 3 Division of Gynecologic Oncology, National Center for Tumor Diseases (NCT), Im Neuenheimer Feld 460, 69120 Heidelberg, Germany; 4 German Cancer Research Center, Im Neuenheimer Feld 280, 69120 Heidelberg, Germany; 5 Department of Pathology, University Hospital Heidelberg, Im Neuenheimer Feld 224, 69120 Heidelberg, Germany

**Keywords:** Metastatic breast cancer, Receptor status discordance, Distant metastasis, Estrogen receptor (ER), Progesterone receptor (PR), Human epidermal growth factor receptor 2 (HER2)

## Abstract

**Background:**

The estrogen receptor (ER), progesterone receptor (PR), and human epidermal growth factor receptor 2 (HER2) statuses are frequently discordant between the primary tumor and metastatic lesions in metastatic breast cancer. This can have important therapeutic implications.

**Patients and methods:**

In all, 541 patients with available receptor statuses from both primary tumor and metastatic lesion treated at Heidelberg and Tuebingen University Hospitals between 1982 and 2018 were included.

**Results:**

Statistically significant discordance rates of 14% and 32% were found for ER and PR. HER2 status was statistically insignificantly discordant in 15% of patients. Gain in HER2 positivity was associated with an improved overall survival, whereas loss of HR positivity was associated with worse overall survival. Antiendocrine treatment differed in 20% of cases before and after biopsy and HER2-directed treatment in 14% of cases.

**Conclusions:**

Receptor statuses are discordant between primary tumor and metastasis in a considerable fraction of patients with metastatic breast cancer. Next to a highly presumed predictive value with respect to efficacy of endocrine and HER2-targeted therapy, discordance seems to provide prognostically relevant information. Where feasible, metastatic lesions should be biopsied in accordance with current guidelines.

**Electronic supplementary material:**

The online version of this article (10.1007/s10549-020-05746-8) contains supplementary material, which is available to authorized users.

## Introduction

With 2.1 million new diagnoses predicted for 2018, breast cancer represents the most prevalent type of cancer in women worldwide [1]. Despite new therapeutic agents and improved survival, (metastatic) breast cancer is considered a leading cause of death among women, with about 700,000 deaths in 2018 [1–3]. Inevitably, therefore, the tumor burden must be closely monitored and targeted systemic therapy (chemotherapy, endocrine therapy, targeted therapy, and biological therapy) or local therapy (surgery and radiation therapy) adjusted.

The estrogen receptor (ER), progesterone receptor (PR), and human epidermal growth factor receptor 2 (HER2) statuses of primary breast cancer tissue are used clinically to approximate biological subtypes, to predict outcome, and to guide therapy decisions, especially for endocrine and HER2-targeted regimens [4, 5]. However, numerous studies have shown substantial discordance rates in ER, PR, and HER2 receptor profiles between primary and metastatic tumors. Based on a meta-analysis of 39 studies, Schrijver et al. [6] reported conversion rates of 19.3% for ER, 30.9% for PR, and 10.3% for HER2, respectively. Yeung et al. [7] showed similar findings in a meta-analysis of 47 studies including 3,384 matched primary and metastatic breast cancer cases with median discordance rates of ER, PR, and HER2-expression of 14%, 21%, and 10%, respectively. Furthermore, loss of receptor positivity is associated with poorer prognosis [8, 9]. Indeed, the meta-analysis by Schrijver et al. showed that therapy was changed due to receptor discordance in 14% for ER, 62% for PR, and 67% for HER2, respectively, assuming that patients gaining hormone receptor positivity qualify for endocrine therapy and those gaining HER2 positivity are eligible for HER2-directed therapy [6].

Given the broad evidence for receptor status conversion during tumor progression, NCCN, ESMO, EGTM, and ASCO guidelines congruently recommend re-testing hormone receptors for metastatic lesions where feasible [4, 10–12]. However, it is not known whether tissue sampling of metastatic sites has any significant effect on patient survival or quality of life [13, 14] and whether the biology of the primary or the metastatic lesion(s) should guide therapeutic decisions [11].

Our study retrospectively compared ER, PR, and HER2 receptor profiles in biopsies of primary breast cancer and corresponding metastatic lesions in a large study population to assess individual changes throughout tumor progression and location-specific discordance rates.

## Patients and methods

### Study design and samples

This study includes women aged 18 or older with metastatic breast cancer where expression level data were available for at least one of the receptors—estrogen, progesterone, or human epidermal growth factor receptor 2—for the primary tumor and corresponding metastatic lesion. If more than one metastatic lesion was available, only the lesion first biopsied was evaluated. Patients were enrolled between 1982 and 2018 at the National Centre for Tumor Diseases (NCT) in Heidelberg, the Department of Obstetrics and Gynecology at the University Hospital Heidelberg, and the University Hospital Tuebingen, Germany. This study was approved by the ethics committee of the medical faculty of Heidelberg (S-295/2009) and Tuebingen (270/2014A) University.

### ER, PR, and HER2 assessment

ER, PR, and HER2 receptor status as well as histopathological characteristics and clinical documentation were retrospectively collected from medical records. Analysis of the hormone receptor status were performed at the University Hospital Tuebingen, the University Hospital Heidelberg and in some instances at peripheral hospitals and defined as hormone receptor-positive according to local standards. The tumor was defined as hormone receptor (HR)-positive if the receptor status of either ER or PR was immunohistochemically positive. HER2 status was assessed by immunohistochemistry (IHC) and/or fluorescent in situ hybridization (FISH). According to ASCO guidelines, HER2 status was positive when the ISH score (0, 1+ , 2+ , 3+) was either 3 + or the ISH score was + 2 with positive fluorescence in situ hybridization (FISH) or chromogenic in situ hybridization in addition (CISH) staining [15].

Follow-up and survival status were documented until loss to follow-up or death. Data were censored at the last follow-up.

### Statistical analysis

McNemar’s test was used to compare paired nominal data between the primary tumor and matched metastasis of individual patients. Association of nonpaired nominal data was tested using Fisher’s exact test. The median follow-up was calculated using the reverse Kaplan–Meier method. The log-rank test was used to compare survival distributions. Overall survival was defined as time difference between first diagnosis of any metastasis and death. Patients in whom no event was documented or who were lost to follow-up were censored. Statistical analysis and visualization were performed using R version 3.5.0 with the packages ggplot2 version 3.1.0 and survminer version 0.4.3. The significance level was set to *⍺* < 0.05. Tests were performed in a two-sided fashion.

## Results

Data from 541 patients, 324 from Heidelberg and 217 from Tuebingen, were available for analysis. In all, 105 (20%) patients had already developed metastases at primary diagnosis. The menopausal status at primary diagnosis was available for 243 patients, 81 (33%) of whom were premenopausal. ER status for both primary tumor and metastasis was available from 538, PR status from 536, and HER2 status from 456 patients. The primary tumor was ER-positive in 421 (78%) patients, PR-positive in 385 (72%), and HER2-positive in 92 (20%). The ER, PR, and HER2 status of the metastases was positive in 382 (71%), 275 (51%), and 102 (22%) cases, respectively. The median follow-up was 58 months. A total of 291 deaths were recorded. Further patient and tumor characteristics can be found in Table 1.

**Table 1:** Clinicopathological characteristics by receptor status concordance

		All patients (%)	ER status			PR status			HR status			HER2 status		
			Discordant	Concordant	*p* (Fisher)	Discordant	Concordant	*p* (Fisher)	Discordant	Concordant	*P* (Fisher)	Discordant	Concordant	*p* (Fisher)
Metastases	Synchronous (%)	105 (20)	13 (12)	92 (88)	0.873	32 (31)	73 (70)	0.727	12 (11)	93 (89)	0.745	13 (14)	77 (86)	0.871
	Metachronous (%)	425 (80)	58 (14)	345 (86)		138 (33)	282 (67)		55 (13)	367 (87)		55 (15)	301 (85)	
Histology	NST (%)	305 (75)	41 (14)	263 (87)	1	102 (34)	201 (66)	0.610	44 (15)	260 (86)	0.902	44 (17)	220 (83)	0.489
	ILC (%)	76 (19)	10 (13)	65 (87)		29 (39)	45 (61)		9 (12)	66 (88)		7 (12)	50 (88)	
	Other (%)	26 (6)	3 (12)	23 (86)		8 (31)	18 (69)		3 (12)	23 (89)		2 (8)	22 (92)	
Menopausal status	Premenopausal (%)	81 (33)	16 (20)	65 (80)	0.262	27 (33)	54 (67)	0.884	14 (17)	67 (83)	0.451	11 (17)	53 (83)	0.840
	Postmenopausal (%)	162 (67)	22 (14)	139 (86)		51 (32)	109 (68)		22 (14)	139 (86)		22 (16)	115 (84)	
Grading	1 (%)	20 (4)	3 (15)	17 (85)	0.747	8 (40)	12 (60)	0.639	3 (15)	17 (85)	0.763	2 (11)	16 (89)	0.805
	2 (%)	263 (55)	33 (13)	229 (87)		88 (34)	173 (66)		32 (12)	230 (88)		34 (16)	182 (84)	
	3 (%)	197 (41)	29 (15)	167 (85)		61 (31)	135 (69)		28 (14)	168 (86)		24 (13)	155 (87)	
Location of first biopsy	Bone (%)	114 (21)	12 (11)	101 (89)	0.123	41 (37)	71 (63)	0.312	10 (9)	103 (91)	0.077	13 (14)	82 (86)	0.763
	Liver (%)	112 (21)	17 (15)	95 (85)		40 (36)	72 (64)		16 (14)	96 (86)		13 (14)	83 (87)	
	CNS (%)	34 (6)	9 (27)	25 (74)		13 (38)	21 (62)		9 (27)	25 (74)		6 (21)	23 (79)	
	Other (%)	279 (52)	35 (13)	242 (87)		80 (29)	196 (71)		35 (13)	242 (87)		36 (15)	198 (85)	

### Receptor status concordance

**Fig. 1:**
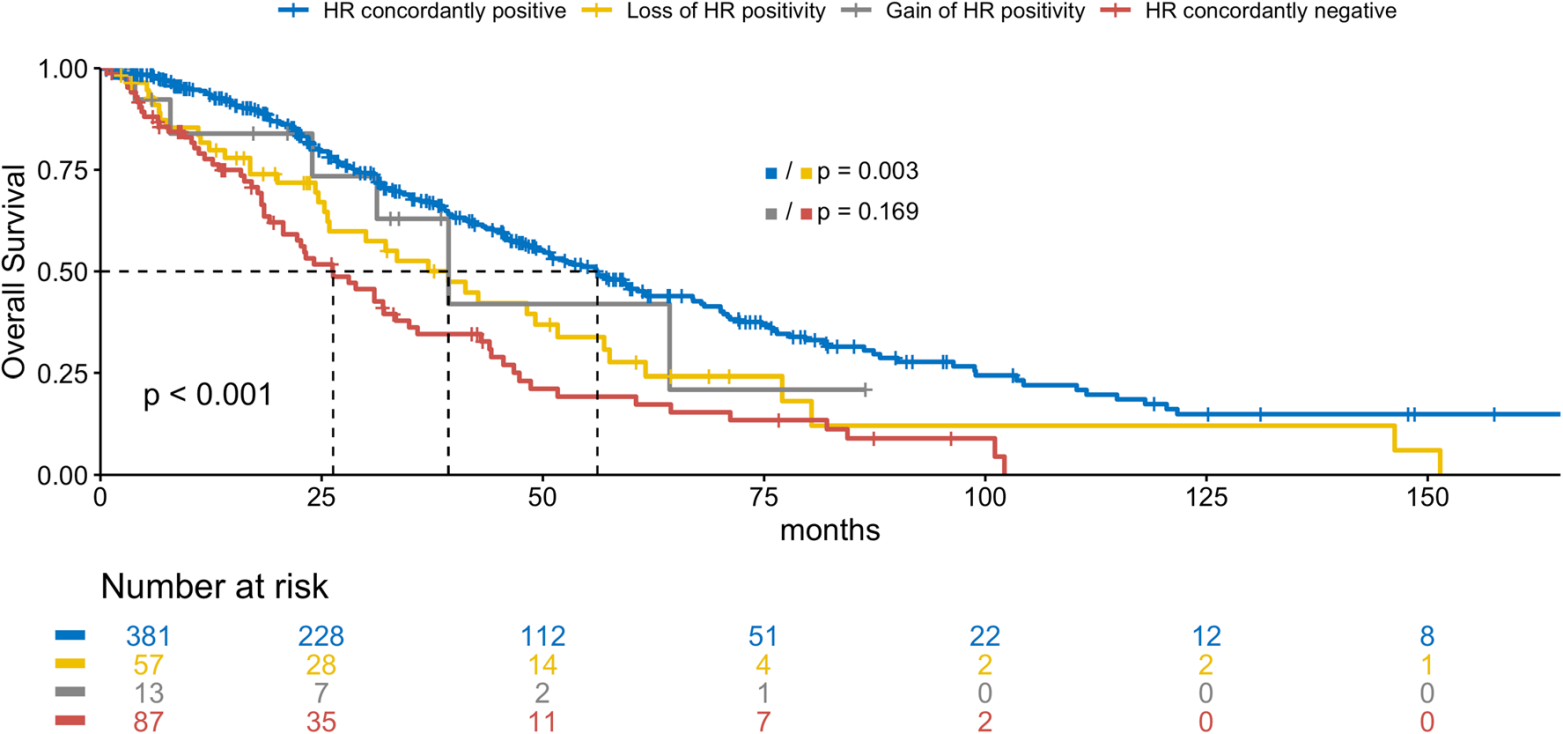
Overall survival by HR status change in months

**Fig. 2:**
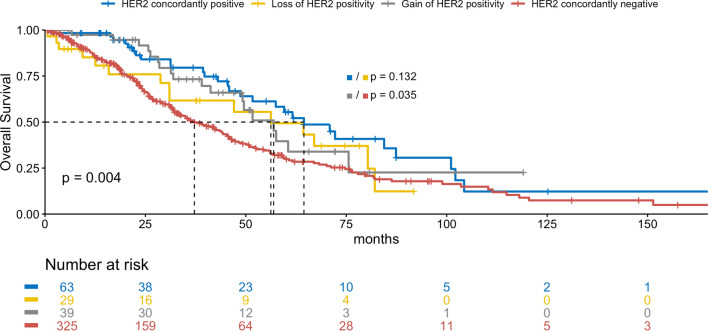
Overall survival by HER2 status change in months

The HR of 70 (13%, *p* < 0.001) patients differed between primary tumor and metastasis, with 13 (19%) patients gaining and 57 (82%) patients losing positivity (Table S1). Among the patients, 73 (14%), 174 (32%), and 68 (15%) had discordant ER, PR, and HER2 statuses in their primary tumor and metastasis, respectively, indicating statistically significant discordance between ER and PR status, but not HER2 status (*p* < 0.001, *p* < 0.001, and *p* = 0.225, Tables S2–4). Of these discordances, 17 (23%), 32 (18%), and 39 (57%) were gains in receptor positivity and 56 (77%), 142 (82%), and 29 (43%) were losses of ER, PR, and HER2 positivity, respectively. The discordance was not statistically significantly different between the locations of metastatic biopsy (Table 1). Loss of HR positivity but not HER2 positivity was associated with a significantly worse OS (HR: median OS 39.3 vs. 56.2 months, *p* = 0.003; HER2: median OS 56.2 vs. 64.5 months, *p* = 0.132), whereas gain of HER2 but not HR positivity was associated with a significantly better OS (HER2: median OS 56.9 vs. 37.2 months, *p* = 0.035; HR: median OS 39.3 vs. 26.3 months, *p* = 0.169, Figs. 1 and 2).

### Antiendocrine and HER2-directed treatment

Data on antiendocrine treatment and HER2-directed therapy before and after biopsy of the first metastasis as well as HR status of both primary tumor and first metastasis were available in 451 and 393 cases, respectively. Antiendocrine treatment changed in 88 (20%, Table 2) and HER2-directed treatment in 55 (14%, Table 3) of cases.

**Table 2:** Antiendocrine treatment by change of hormone receptor status in metastatic biopsy

		Antiendocrine treatment			
		Before and after biopsy	Only before biopsy	Only after biopsy	None
HR Status	Concordantly positive (%)	242 (76)	42 (13)	30 (9)	5 (2)
	Loss of positivity (%)	13 (29)	26 (58)	0 (0)	6 (13)
	Gain of positivity (%)	0 (0)	0 (0)	7 (64)	4 (36)
	Concordantly negative (%)	0 (0)	0 (0)	1 (1)	75 (99)

**Table 3:** HER2-directed therapy by change of HER2 status in metastatic biopsy

		HER2-directed therapy			
		Before and after biopsy	Only before biopsy	Only after biopsy	None
HER2 status	Concordantly positive (%)	30 (58)	9 (17)	11 (21)	2 (4)
	Loss of positivity (%)	2 (10)	10 (50)	2 (10)	6 (30)
	Gain of positivity (%)	1 (3)	0 (0)	22 (61)	13 (36)
	Concordantly negative (%)	0 (0)	0 (0)	1 (0)	284 (100)

## Discussion

The choice of targeted therapy for patients with metastatic breast cancer is guided by ER, PR, and HER2 status of the primary tumor, despite evidence indicating that hormone receptor status may change during tumor progression [4, 6, 11]. Discordant receptor expression between primary tumor and metastatic site is prognostic [16–18] and might be clinically relevant if changes in hormone receptor status go unnoticed. Antiendocrine or HER2-directed therapy might be ineffective, with the additional cost of side effects if the metastatic tumor loses its receptor positivity, whereas potential effective targeted therapy might be withheld if gain of receptor positivity remains undetected. However, conversion rates vary to a great extent in the literature. Furthermore, there is disagreement concerning an association of such changes with outcome and clinical implications.

In our study, we found that clinically used biomarkers were highly unstable between the primary tumor and the metastatic lesion. ER, PR, and HER2 status changed in 14%, 32%, and 15%, respectively. Although the change of HER2 status was not statistically significant, the percentage of discordant patients is clinically meaningful. These discordance rates are similar to the pooled random effects percentages of 19%, 31%, and 10% that Schrijver et al. reported [6]. However, in contrast to previous reports [6], we were unable to see any significant difference between site-specific discordance rates.

Patients who lost HR positivity had a significantly poorer prognosis than concordantly receptor-positive patients. Gain of HER2 positivity was associated with a significantly more favorable prognosis than concordantly negative receptor status which may reflect benefit from adjustment of therapy. This finding is in line with prior studies, underlining the importance of reassessing receptor status in the metastatic setting [8, 9, 19–21].

There are several conceivable explanations for a discordant receptor status in breast tumors, including technical and analytical variability in immunohistochemical analysis, a heterogeneous tumor biology, and biological evolution.

Several studies reported varying concordance of results between the surgical specimen and core needle biopsy of ER (77.8 to 99%), PR (69 to 97%), and HER2 (64 to 97%), respectively, with particular emphasis placed on a general tendency for higher discrepancies in PR status [22–27]. In addition to substantial improvement in receptor measurement accuracy, interlaboratory variation still exists in the assignment of receptor status [28, 29]. The choice of method (such as IHC and RT-PCR), the assay method (dual-antibody vs single-antibody ER assay), as well as sample processing (e.g., decalcification reduced staining intensity especially in bone metastasis) may yield to discordant results [29–31]. Moreover, the cut-off for ER/PR positivity may vary when histology results from different pathologists are compared and have changed over the last decades. Nevertheless, it is unlikely that receptor status conversion in our cohort is solely attributable to technical issues, as differences in OS depending on the ER and PR are biologically meaningful observations given the superior prognosis of hormone receptor-positive breast cancers [32].

Breast cancers are genomically and transcriptomically heterogeneous tumors [33–36]. This intratumoral heterogeneity is also observed for ER expression [16]. Additionally, different metastatic locations seem to activate different gene expression profiles within metastatic cells [37].

As tumors are heterogeneous and sequential biopsies are invasive procedures, increasing focus is being put on liquid biopsies. Here, sequential sample collection is not difficult and technological advances such as the circulating tumor cell detection or the characterization of cell-free tumor DNA in blood are promising approaches [38, 39]. With continuous measurement, these biomarkers could represent current systemic tumor burden, monitor evolving tumor biology in real time, assess treatment efficacy, and, thus, guide therapy more comprehensively [40–42].

In our collective, antiendocrine treatment was subject to change in 20% of patients and HER2-directed treatment in 14% of patients before and after metastasis biopsy. This is in line with previously reported fractions of 18–57% and 7–50% in other cohorts ( [43–47]). Concluding, it is clinically relevant to detect changes in receptor status in the metastatic setting in order to tailor therapeutic interventions according to present tumor manifestation.

The validity of our data is limited and should be interpreted with caution, as in some cases more than one metastasis and/or local recurrences were subjected to biopsy. Moreover, in patients where treatment was carried out in closer to home clinics, therapeutic measures recorded here were mere recommendations, the adherence to which was not recorded. Therefore, causality of metastatic receptor status change and accordingly treatment change as documented in our dataset cannot be established. Moreover, Trastuzumab only received approval for treatment of metastatic breast cancer in 2000 and for adjuvant treatment in patients with early breast cancer in 2006 in Germany. Our dataset, however, also includes patients treated before those dates, which therefore introduces another confounding variable [48].

Further limitations of our study are the retrospective design and the decentralized determination of receptor status. Thus, for some patients immunohistochemical analysis of the primary tumor and the metastasis was carried out in different laboratories. Furthermore, biopsies were collected and analyzed over a period of about 15 years, during which cut-off values for hormone receptor status were lowered. However, this approach reflects clinical reality, especially as endocrine or HER2-directed treatment was chosen due to the respective definition of ER-/PR and HER2 positivity.

## Conclusions

We found that 13% and 15% of 543 patients from Heidelberg and Tuebingen University Hospitals with metastatic breast cancer had a discordance of HR and HER2 status between their primary tumor and metastasis. Antiendocrine treatment and HER2-directed treatment changed in 20% and 14%, respectively. As receptor conversion influences endocrine and HER2-directed therapy decision and significantly impacted OS these results are of high clinical relevance. Hence, in accordance with current guidelines, our results confirm that biopsy of metastatic tissue should be pursued wherever feasible.

## Electronic supplementary material

Below is the link to the electronic supplementary material.

Supplementary file1 (DOC 77 kb)
