# Supplementary material for: Estrogen, progesterone, and human epidermal growth factor receptor 2 discordance between primary and metastatic breast cancer
Source: Breast Cancer Res Treat. 2020 Jul 1;183(1):137–44. doi: 10.1007/s10549-020-05746-8 (PMC7375990; doi:10.1007/s10549-020-05746-8)
Supplement: Supplementary file 1 — Supplementary file1 (DOC 77 kb) [file 10549_2020_5746_MOESM1_ESM.doc]

Table S1 Hormone receptor status concordance.

|  |  | **Metastasis** | |  |
| --- | --- | --- | --- | --- |
|  |  | HR- | HR+ | *p* (McNemar) |
| **Primary** | HR- (%) | 87 (87) | 13 (13) | <0.001 |
|  | HR+ (%) | 57 (13) | 381 (87) |  |

Table S2 Estrogen receptor status concordance.

|  |  | **Metastasis** | |  |
| --- | --- | --- | --- | --- |
|  |  | ER- | ER+ | *p* (McNemar) |
| **Primary** | ER- (%) | 100 (86) | 17 (15) | <0.001 |
|  | ER+ (%) | 56 (13) | 365 (87) |  |

Table S3 Progesterone receptor status concordance.

|  |  | **Metastasis** | |  |
| --- | --- | --- | --- | --- |
|  |  | PR- | PR+ | *p* (McNemar) |
| **Primary** | PR- (%) | 119 (79) | 32 (21) | <0.001 |
|  | PR+ (%) | 142 (37) | 243 (63) |  |

Table S4 Human epidermal growth factor 2 receptor status concordance.

|  |  | **Metastasis** | |  |
| --- | --- | --- | --- | --- |
|  |  | HER2- | HER2+ | *p* (McNemar) |
| **Primary** | HER2- (%) | 325 (89) | 39 (11) | 0.225 |
|  | HER2+ (%) | 29 (32) | 63 (89) |  |
